# Scrutinizing assortative mating in birds

**DOI:** 10.1371/journal.pbio.3000156

**Published:** 2019-02-21

**Authors:** Daiping Wang, Wolfgang Forstmeier, Mihai Valcu, Niels J. Dingemanse, Martin Bulla, Christiaan Both, Renée A. Duckworth, Lynna Marie Kiere, Patrik Karell, Tomáš Albrecht, Bart Kempenaers

**Affiliations:** 1 Department of Behavioural Ecology and Evolutionary Genetics, Max Planck Institute for Ornithology, Seewiesen, Germany; 2 Behavioural Ecology, Department of Biology, Ludwig Maximilians University of Munich, Planegg-Martinsried, Germany; 3 NIOZ Royal Netherlands Institute for Sea Research, Department of Coastal Systems and Utrecht University, Den Burg, the Netherlands; 4 Department of Ecology, Faculty of Environmental Sciences, Czech University of Life Sciences Prague, Kamýcká, Prague, Czech Republic; 5 Conservation Ecology Group, Groningen Institute for Evolutionary Life Sciences, University of Groningen, Groningen, the Netherlands; 6 Department of Ecology and Evolutionary Biology, University of Arizona, Tucson, Arizona, United States of America; 7 Center for the Study of Biodiversity and Conservation (CIByC), Autonomous University of the State of Morelos, Cuernavaca Morelos, Mexico; 8 Institute of Ecology, Department of Evolutionary Ecology, Universidad Nacional Autónoma de México, Mexico City, Distrito Federal, Mexico; 9 Bioeconomy Research Team, Novia University of Applied Sciences, Raseborgsvägen, Ekenäs, Finland; 10 Institute of Vertebrate Biology of the Czech Academy of Sciences, Brno, and Faculty of Science, Charles University, Prague, Czech Republic; The Australian National University, AUSTRALIA

## Abstract

It is often claimed that pair bonds preferentially form between individuals that resemble one another. Such assortative mating appears to be widespread throughout the animal kingdom. Yet it is unclear whether the apparent ubiquity of assortative mating arises primarily from mate choice (“like attracts like”), which can be constrained by same-sex competition for mates; from spatial or temporal separation; or from observer, reporting, publication, or search bias. Here, based on a conventional literature search, we find compelling meta-analytical evidence for size-assortative mating in birds (r = 0.178, 95% CI 0.142–0.215, 83 species, 35,591 pairs). However, our analyses reveal that this effect vanishes gradually with increased control of confounding factors. Specifically, the effect size decreased by 42% when we used previously unpublished data from nine long-term field studies, i.e., data free of reporting and publication bias (r = 0.103, 95% CI 0.074–0.132, eight species, 16,611 pairs). Moreover, in those data, assortative mating effectively disappeared when both partners were measured by independent observers or separately in space and time (mean r = 0.018, 95% CI −0.016–0.057). Likewise, we also found no evidence for assortative mating in a direct experimental test for mutual mate choice in captive populations of Zebra finches (r = −0.020, 95% CI −0.148–0.107, 1,414 pairs). These results highlight the importance of unpublished data in generating unbiased meta-analytical conclusions and suggest that the apparent ubiquity of assortative mating reported in the literature is overestimated and may not be driven by mate choice or mating competition for preferred mates.

## Abstract 2

Human mate choice is characterized by assortative mating (‘like attracts like’) and similarity of partners is also often reported for birds. A meta-analysis of published and previously unpublished datasets shows that the reported assortative mating in birds may mostly reflect biases in estimation rather than mate choice.

## Author summary

Research on mate choice in birds has attracted much attention, partly because many birds form monogamous pair bonds like humans do. Human mate choice is characterized by the phenomenon of “like attracts like,” meaning that partners resemble each other in multiple ways (“assortative mating”). Assortative mating is also frequently reported for birds, but it is unclear whether this in turn implies that birds also have preferences for a similar partner. Here, we show that a range of methodological issues may provide a simpler and more accurate explanation for the frequent observation of assortative mating in birds. First, studies that report assortative mating may achieve greater visibility than studies that yield no such finding. Hence, the scientific literature may be biased toward positive results. Second, in field studies, it is logistically impossible to measure all birds accurately and under standardized conditions. Hence, fluctuations in, for instance, environmental conditions may induce a spurious similarity between partners when these are measured together in space or time. After accounting for such methodological issues, we conclude that mate preferences for a similar partner may be less common than previously thought.

## Introduction

Members of a pair often resemble each other. For instance, in humans, partners have similar political attitudes [1, 2], level of education [3, 4], and body height [4–6]. In fact, assortative mating appears to be common across all animal taxa and across all phenotypic traits that have been investigated [7]. However, in most cases, the underlying processes that lead to mate similarity remain unclear.

Similarity of pair members, quantified as the strength of the correlation between their trait values, may arise via at least three biological mechanisms. (1) Mate choice and mating competition; one or both sexes may prefer phenotypes similar to their own (“like attracts like”). This may lead to the more frequent formation and enhanced stability of assortative pair bonds. (2) Spatial and temporal autocorrelation; individuals with different phenotypes may be separated in space and time, such that at the population level, even random mating would lead to partner similarity (“like meets like”). For instance, in high-quality habitats, individuals may grow larger than in poor habitats. If individuals from different habitats are less likely to meet (“nonpanmixis”), e.g., because of low mobility [8], a population-wide pattern of assortative mating may arise in the absence of choice for an assortative partner. Similarly, in migratory species, individuals that resemble each other in particular traits may have a higher probability to form a pair simply because they arrive at the breeding grounds closer in time (e.g., older individuals might arrive earlier, leading to assortative mating for age [9]). (3) Phenotypic changes over time; females and males may mate randomly for a certain phenotype but become similar to their partner over time (“become alike”) [10]. For instance, in humans, a positive correlation in body mass between couples may arise because they share the same food [11, 12]. Because the three biological mechanisms can act together and can also interact with other processes, their relative importance may be difficult to tease apart [13, 14]. Furthermore, if testing assortative mating that comes about via mate choice and mating competition is the goal of the study, then the other “biological” mechanisms could be considered as confounds [15]. Also, it should be noted that not all mechanisms may be relevant for all phenotypes (e.g., “becoming alike” does not apply to assortative mating with regard to age).

Besides the influence of biological processes [16], estimates of the strength of assortative mating can be confounded by three main methodological issues. (1) Observer bias; data sets often consist of measurements from multiple observers and may be taken over long periods. This introduces heterogeneity and temporal autocorrelation in the data because observers can differ in their measurements [17], and they may unconsciously change their measuring technique over time. Hence, spurious trait correlations between pair members may arise when pair members are typically measured by the same observer and on the same day (see our simulation in S1 Fig). Such methodological heterogeneity may be difficult to distinguish from biological heterogeneity in space and time. (2) Reporting bias; estimates found in the literature will be inflated when statistically significant estimates are more likely reported than nonsignificant ones [18, 19]. (3) Search bias; if a meta-analysis is based on a literature search with keywords like “assortative,” the strength of assortative mating may be overestimated because it is less likely null results are mentioned in the abstract of a publication [20]; hence, such searches may preferentially yield a subset of studies that have detected significant assortative mating. Similarly, when screening relevant publications, taking estimates from related studies that are being cited may also discriminate against null findings because studies with null findings tend to get cited less often than studies with significant and, hence, typically larger effects [21].

Assortative mating is often investigated with a focus on mate choice and mating competition [22, 23]. However, a systematic review of assortative mating in arthropods [13] suggests that mechanisms other than “mating preferences for phenotypic similarity” are predominantly responsible for the mating patterns observed in these invertebrates (such as mate availability and physical constraints). Whether mate choice and mating competition or other processes underlie assortative mating in vertebrates such as birds is unclear, particularly because birds often form strong social pair bonds for which the compatibility of partners may determine reproductive success [24].

Here, we quantify the strength of assortative mating in birds and assess how assortative mating estimates change with increasing control of the confounding factors discussed above. We then discuss biological implications of our findings and propose ways to minimize confounding effects if the aim is to investigate assortative mating due to mate choice and mating competition. For practical reasons (data availability), we focus primarily on assortative mating for size in birds, yet our approach is relevant for most phenotypic traits and taxa. In birds, body size is measured and reported in a variety of ways (body mass, wing length, tarsus length, bill length, body length, and first principal component of different measures). Thus, our measure of body size includes all size-related morphological measures but not measures of ornament size (e.g., plumage patch area or ornamental feather length) and of body condition (e.g., mass residuals over tarsus length), which were categorized separately.

First, we compare published estimates of the strength of assortative mating with estimates from our previously unpublished data from nine long-term field studies that are a sample of the species studied in the literature. This allows assessment of the effect of search and reporting bias, which should only affect the published data set. Second, we use the previously unpublished data set to explore causes of assortative mating patterns that are not due to mate choice and mating competition, such as effects of observer bias and spatial and temporal nonindependence. Finally, we present an analysis of experimental data from five studies of assortative mating in captive Zebra finches, *Taeniopygia guttata* [24, 25]. In these experiments, we took standardized measurements of all birds before randomly allocating them to experimental aviaries. So far, this is the only avian study for which we can estimate the strength of assortative mating among individuals that encountered each other, excluding all known confounding factors.

## Results

### Assortative mating: All traits—published literature

**Fig 1:**
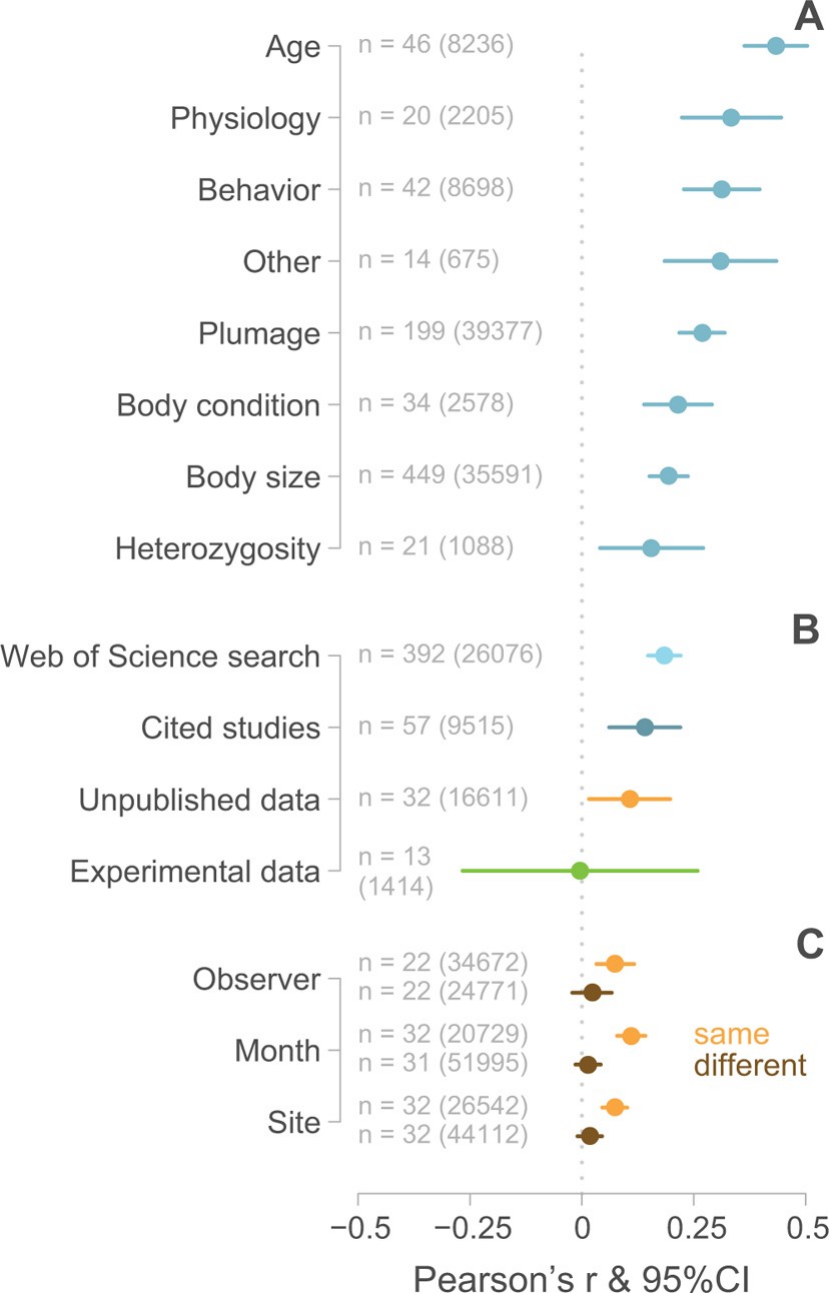
Assortative mating in birds. (A) The magnitude of assortative mating for various traits based on a meta-analysis of the published literature. The data comprise both the “Web of Science search” and the “Cited studies” (see Methods). (B) Strength of assortative mating for size as a function of data source. (C) Strength of assortative mating calculated from previously “Unpublished data” (nine long-term field studies) as a function of measurement context. Dots represent mean Pearson’s correlation coefficients (r), bars the 95% CI (based on S1, S5 and S7 Tables). *n* indicates the number of estimates for a given trait category, followed by the number of pair–trait combinations in parentheses. The dotted line indicates no assortative mating (r = 0), negative r-values indicate disassortative mating, and positive r-values indicate assortative mating. Underlying data for this figure can be found in S4, S5, and S6 Data.

Overall, the published literature showed striking evidence for positive assortative mating across all trait categories (ranging from r = 0.156 to 0.434; none of the 95% CI overlap zero) (Fig 1 and S1 Table). The two random effects, “Study” and “Species,” explained only 9% and 6% of the variance, respectively. Compared to other traits, assortative mating for body size was the second weakest (r = 0.178, 95% CI 0.142–0.215, 83 species, 35,591 pairs), but it was the most frequently studied trait (54% of all estimates).

### Assortative mating for size: Previously unpublished field studies

The previously unpublished data from nine long-term field studies also showed a clear yet weaker tendency for positive assortative mating by size, whereby the magnitude depended on how the data were analyzed (ranging from r = 0.067 to 0.103) (S2 Fig and S2, S3 and S4 Tables).

When repeatedly measured individuals were represented by a randomly selected single measure (i.e., model of “random alignment” of male to female measure), the strength of assortment was weak (mean r = 0.067, 95% CI 0.042–0.093, *n* = 16,545 pair–trait combinations) (S2 Fig and S2 Table). When average measures per individual were used (“average model”), estimates of assortment were only slightly higher (mean r = 0.080, 95% CI 0.054–0.105, *n* = 16,545) (S2 Fig and S3 Table). Finally, when using the male and female measures taken closest to the presumed time of pair formation (“nearest model”), the estimate of assortative mating was highest (mean r = 0.103, 95% CI 0.074–0.132, *n* = 16,611) (S2 Fig and S4 Table). The estimates from the “nearest” model were significantly higher than those from the “random alignment” model (paired *t* test for 32 species–traits, t_31_ = 4.50, *P* = 0.0001) and higher than estimates from the “average” model (t_31_ = 3.32, *P* = 0.002).

### Effects of observer, time, and space on estimates of assortative mating

In the previously unpublished data set, levels of apparent assortative mating were significantly higher when measurements on the two members of a pair had been taken by the same observer (r = 0.075, 95% CI 0.033–0.118, t = 3.45, *P* = 0.0006, *n* = 22 estimates, 34,672 pair–trait combinations) than when measurements came from different observers (r = 0.023, 95% CI −0.022–0.067, t = 1.01, *P* = 0.3, *n* = 22 estimates, 24,771 pair–trait combinations; t_same versus different observer_ = 2.48, *P* = 0.01) (Fig 1 and S5 Table: model 4).

Similarly, estimates for assortative mating for size were significantly higher when measurements on the two members of a pair had been taken within the same month (within 30 days: r = 0.110, 95% CI 0.079–0.142, t = 6.92, *P* < 0.0001, *n* = 32 estimates, 51,995 pair–trait combinations) than when the partners had been measured more than 30 days apart (r = 0.014, 95% CI −0.013–0.041, t = 0.98, *P* = 0.3, *n* = 31 estimates, 20,729 pair–trait combinations; t_same versus different time_ = 5.7, *P* < 0.0001) (Fig 1 and S5 Table: model 5).

Finally, estimates of size-assortative mating were significantly higher when partners had been measured at the same site (0.073, 95% CI 0.046–0.100, t = 5.30, *P* < 0.0001, *n* = 32 estimates, 26,542 pair–trait combinations) than when they were measured at different sites (0.017, 95% CI −0.009–0.044, t = 1.28, *P* = 0.2, *n* = 32 estimates, 44,112 pair–trait combinations; t_same versus different location_ = 3.90, *P* < 0.0001) (Fig 1 and S5 Table: model 6).

### Assortative mating for size: Experimental study

Data from the five experiments on Zebra finches showed an overall size-assortative mating close to zero (r = −0.020, 95% CI −0.148–0.107; weighted mean of 13 estimates based on 1,414 pair–trait combinations) (S6 Table and S8 Data). Note that the statistical power for detecting an effect of r = 0.20 was >0.99 for each of the three size phenotypes (mass, tarsus, and wing length).

### Effect of data source on estimates of size-assortative mating

The strength of assortative mating decreased with increasing control for confounding factors from published (Web of Science search: r = 0.184; Cited studies: r = 0.140) to previously unpublished (r = 0.107) to experimental data (r = −0.004). However, in such a joint model, CIs around the estimates were relatively wide despite high sample sizes (Fig 1 and S7 Table). The random effect “Study” explained 7% of the variance, while the other two random effects (“Species” and “Trait”) explained zero variance (S7 Table).

### Effect of sample size on estimates of assortative mating strength

**Fig 2:**
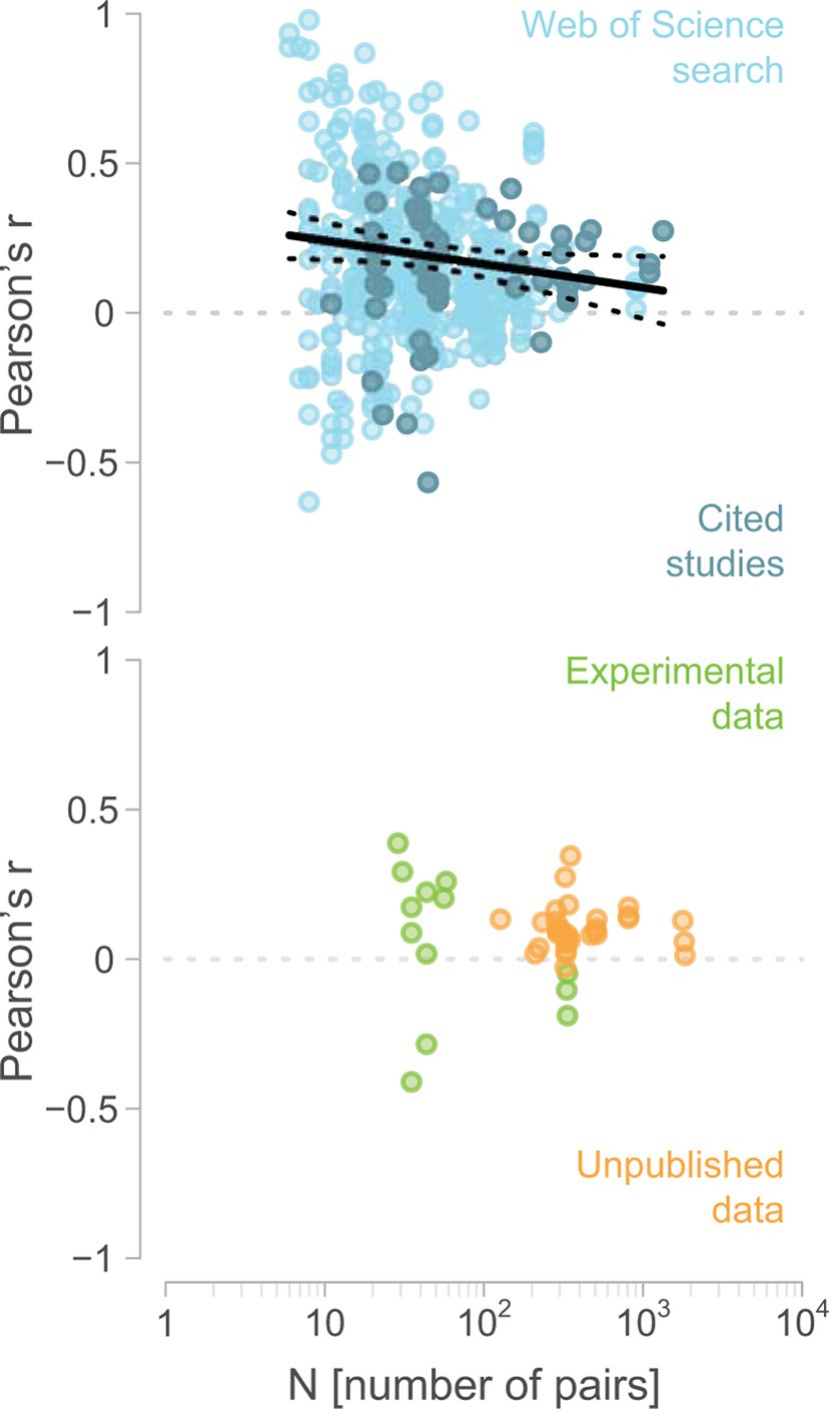
Strength of assortative mating for body size in relation to sample size. Each dot represents a single estimate of assortative mating for a phenotypic trait, its colour the type of estimate (light blue, Web of Science search; dark blue, Cited studies; green, Experimental data; orange, previously Unpublished data). The black regression line refers to all published estimates (Web of Science search plus Cited studies), and dashed lines indicate the 95% CI. Underlying data can be found in S7 Data.

Estimates of assortative mating from the literature data declined significantly with increasing sample size (test for asymmetry in the funnel plot t = −2.76, *P* = 0.006, *n* = 449) (Fig 2). According to the estimated regression line (Fig 2), correlation coefficients from the “Published data” reached the same magnitude as those from the previously “Unpublished data” at a sample size of *n* = 822 pairs.

## Discussion

Our meta-analysis of published estimates of assortative mating shows strong evidence for positive assortative mating in birds across different phenotypes (Fig 1). This is consistent with a recent meta-analysis of assortative mating across the whole animal kingdom [7]. However, using data sets of body size from different sources as an example, our study reveals that seemingly robust effects may disappear when controlling for multiple non-mate–choice mechanisms (Fig 1). Given the higher flexibility in other phenotypic traits, such as physiology and behavior, this problem is likely pervasive. Hence, our study questions the ubiquity and importance of assortative mating due to mate choice and mating competition and suggests that the meta-analytical results cannot be taken as evidence for mate choice for a similar partner (“like attracts like”). In the following, we discuss the effects of each confounding factor, and when possible, we suggest ways to avoid the bias.

### Evidence for search and reporting bias

A recent analysis of assortative mating across the animal kingdom [7] did not find clear evidence for publication bias, as indicated by significant asymmetry in the funnel plot, while in our data set of published studies, the funnel plot asymmetry was statistically significant (Fig 2). This could indicate either that publication bias is limited or that tests for asymmetry in the funnel plot are sometimes inefficient in detecting it [26]. Publication bias is expected because (a) most studies emphasize positive findings rather than null results [27] despite statistical power being low (selective reporting [28]), (b) incomplete reporting of nonsignificant outcomes is widespread [29], and (c) the few meta-analyses that compared published and unpublished effect sizes (including this one) found that published effects were larger than unpublished ones (Fig 1) (e.g., [30–32]). In this context, the increased availability of open access data [33] might allow an unbiased quantification of effect sizes (particularly when the published data were part of a study addressing a different question), which might help to put the existing knowledge into perspective.

### Evaluating observer bias

In field studies, it is practically impossible to implement the optimal measuring design of experimental studies (all individuals measured in random order by the same observer in the same environment on the same day before mate choice starts). This means that multiple observers, multiple environments, and multiple time points introduce heterogeneity into the data, which is partly of methodological and partly of biological origin. Such heterogeneity may (1) partly blur existing patterns of assortment due to sexual selection, thereby leading to underestimation, and (2) induce spurious correlations between pair members when these are often measured by the same observer and close in time and space, leading to overestimation of assortment due to sexual selection. As our simulation shows, the effect of an overestimation of assortative mating clearly exceeds the effect of underestimation when (1) levels of true assortment are relatively low and (2) a large fraction of the pair members are measured by the same observer (see S1 Fig). In our empirical data, we found the expected difference between the “same” versus “different” observer conditions, as the simulation showed (Fig 1), and conclude that our “nearest” model tended to overestimate true levels of assortment because for 80% of the pairs, the partners had been measured by the same observer (S1 Fig).

When it is not practically feasible that all measurements are taken by a single observer, observer bias can be controlled for by calculating correlations between pair members after statistically removing observer effects (see [34] for a solution). Measurement error may also fluctuate in time and thereby induce temporal autocorrelation and, hence, an apparent similarity of pair members (see next paragraph). Finally, observers may have preconceptions about assortative mating, such that measurements suffer from confirmation bias [35]. This is perhaps less likely for data sets that were collected without hypotheses about assortative mating in mind (such as ours). Preconception bias can be solved by blinding observers to the hypothesis of interest [36]; this is already ensured when the members of a pair are measured by independent observers or at different points in time.

### Assortative mating patterns due to temporal and spatial autocorrelation

Our results show that the estimated strength of size-assortative mating is higher when individuals are measured within the same month or at the same site (Fig 1). Such results may arise for both biological and technical reasons. For example, measures of plumage coloration may show temporal autocorrelation because plumage color gradually changes after moult due to wear or because of changes in the white balance used for calibration of hand-held photo spectrometers [37]. Also, depending on the research question, effects that induce temporal and spatial autocorrelation can be of biological interest (e.g., for ecologists), or it can be a confounding factor (e.g., if the interest lies in studying mate choice and mating competition). In the latter case, one can use bivariate mixed models to disentangle the confounding effects of space or time, such that the correlation at the pair level will more closely reflect assortative mating due to mate choice and mating competition [34, 38].

### Assortative mating due to mate choice and mating competition or other processes

We found that mate choice is an unlikely driver of size-assortative mating in birds (Fig 1). The observed effect sizes were much lower than, for instance, the level of assortative mating for body height in humans (r = 0.23, [6]), for which body height is only of secondary importance for mate choice [39]. To robustly examine whether assortative mating arises from (mutual) mate choice and mating competition rather than from other processes (e.g., “like meets like” or “become alike”), an experimental approach is needed in which all individuals can be measured before they mate (see “experimental study” in Methods). Further, such an experimental approach is inevitably needed when groups of individuals (potential mates) are spatially and temporally segregated (nonpanmixis). This is because evidence for the role of mate choice requires knowledge about the potential partners available during pair formation (who encountered whom). Also, an experimental approach allows disentangling scenarios in which separation in space or time according to certain phenotypic traits is already a consequence of choosiness (i.e., individuals separate in space because they have already been rejected, e.g., during courtship displays).

Often, however, an experimental approach is not feasible. In that case, a targeted analysis of binary mating decisions observed in the field (e.g., evidence for rejection versus acceptance of an individual) may provide some clues. In the end, knowledge of a study system and the consideration of possible confounds (“like meets like” and “become alike”) may help separating mate choice and mating competition from other causal factors.

### Conclusions

Assortative mating for certain phenotypic traits may arise from various biological processes and methodological issues. We thus argue for careful consideration of mechanisms and confounding effects. Our results show (a) that assortative mating can arise through many processes other than mate choice and mating competition [8, 13, 40] and (b) that it might be less strong than suggested by meta-analyses of the published literature [7]. In this respect, the value of unpublished data sets deserves greater appreciation.

## Methods

### Ethics statement

This study was carried out under the license of Max Planck Institute for Ornithology Animal Care; the Animal Experimentation Committee of the University of Groningen; Mexican authorities, including the administration of Isla Isabel National Park, the National Commission for Protected Natural Areas (CONANP), and the Secretary of Environment and Natural Resources (SEMARNAT, permit number SGPA/DGVS/08333/10). Field protocols of Barn swallow were noninvasive and adhered to the laws and guidelines of the Czech Republic (Czech Research Permit number 6628/2008-10001). All protocols were approved by the Animal Care and Use Committees at the Czech Academy of Sciences (041/2011 and 09/2015) and Charles University in Prague (4789/2008-30). The procedures required to collect the data of Tawny owl, which all fall under the ringing licenses of the authors, as provided by the relevant local authorities. Data collection on Western bluebird was carried out in accordance with the recommendations and guidelines approved by University Institutional and Animal Care and Use Committees and under state and federal permitting guidelines.

### Published data

#### Literature search and inclusion criteria

On the 12th of October 2018, we searched for published literature on assortative mating in birds from all data bases in Web of Science with the keywords “birds” and “*assortative mating” (which also covers the term “disassortative mating”). This resulted in 522 hits, of which 165 studies focused on assortative mating within populations (as opposed to studies on the mixing of two defined populations, e.g., in hybrid zones). The 165 studies contained 722 estimates of the strength of assortative mating for any phenotype from 127 species. We refer to these data as “Web of Science search.”

Next, we identified studies missed in the “Web of Science search” by screening the introduction and discussion sections of the 165 publications mentioned above. Of the 71 additional studies identified, 34 concerned assortative mating within populations. These 34 studies contained 103 estimates of assortative mating from 31 species. We refer to this data set as “Cited Studies.”

#### Data extraction and categorization

From both data sets, we extracted the Pearson’s correlation coefficient (r) as an estimate of the strength of assortative mating between pair members. If “r” was missing, we calculated it from the following three test statistics.

F-test with a single numerator degree of freedom and denominator degrees of freedom (df): [41]

$$\text{r}\;=\sqrt{\frac{F\left(1,-\right)}{F\left(1,-\right)+df}}$$

χ^2^ statistic with one degree of freedom and sample size (*n*): [41]

$$\text{r}\;=\sqrt{\frac{\chi 2}{n}}$$

For studies in which the strength of assortment was reported in 2 x 2 contingency tables (e.g., two different plumage types), we calculated r following Nakagawa and Cuthill [42]:

$$\text{r}=\frac{\text{AD}-\text{BC}}{\sqrt{\left(\text{A}+\text{B}\right)\left(\text{C}+\text{D}\right)\left(\text{A}+\text{C}\right)\left(\text{B}+\text{D}\right)}}$$

in which A, B, C, and D represent the observed cell frequencies, and *n* = A + B + C + D = the total sample size.

To avoid pseudoreplication, we checked multiple studies on the same species (especially those from the same research group) and excluded redundant estimates from the same population and same period (*n* = 3), giving priority to the estimate based on the largest sample size.

We classified the phenotypic traits for which assortative mating had been reported into one of eight trait categories: body size (*n* = 449 estimates), body condition (*n* = 34), plumage coloration (*n* = 199), age (*n* = 46), behavior (*n* = 42), physiology (*n* = 20), heterozygosity (*n* = 21), and other (*n* = 14). Here, we focus on the best-documented assortment by body size traits.

#### Estimating assortative mating

To estimate assortative mating, Pearson’s r − weighed by sample size ([*n −* 3]^0.5^, in which *n* is the number of pairs [42]) was modeled as the dependent variable, with “Type of trait” as a fixed effect (factor with eight levels) and “Species” and “Study” (i.e., publication) as random effects (lme4 package in R 3.1.1 [43, 44]). We removed the intercept to obtain parameter estimates and 95% CI for each trait.

### Previously unpublished data from long-term field studies

#### Selection of studies

To obtain data from comparable field studies that have not gone through the filtering steps of publication and detection via search terms or citation, we used data from our own long-term field studies and contacted the researchers who run such studies. All but one agreed to provide the raw data, yielding nine data sets from eight different species and from 4–38 years of study (see S1 Text for details). The studies were chosen based on personal contacts, independent of knowledge about mate choice, but with the aim to include both nonpasserines (*n* = 3) and passerines (*n* = 5; Table 1).

**Table 1:** Overview of the previously “Unpublished data” from nine long-term field studies.

								Data availability							
Species name	Abbreviation	Country	Ref.	Study duration (years)	*n* unique pairs^1^	% multiple measurements^2^	*n* combi-nations^3^	Culmen	Mass	Ulna	Tail	Tarsus	Wing	Primary	Head
Barn swallow *Hirundo rustica*	BS	Czech Republic	[45, 46]	6	235	63.0%	2.7		Y		Y	Y	Y		
Blue-footed booby *Sula nebouxii*	BB	Mexico	[47, 48]	4	510	20.5%	1.4	Y	Y	Y					
Blue tit *Cyanistes caeruleus*	BT_K	Austria	[49, 50]	9	332	90.6%	11.8		Y			Y	Y		
Blue tit *Cyanistes caeruleus*	BT_W	Germany	[51]	7	511	81.5%	5.5		Y			Y		Y	
Great tit *Parus major*	GT	Germany	[52]	6	814	66.0%	3.4		Y			Y		Y	
Pied flycatcher *Ficedula hypoleuca*	PF	Holland	[53]	9	1,832	76.7%	4.1		Y			Y		Y	
Semipalmated sandpiper *Calidris pusilla*	SS	USA	[54, 55]	7	325	49.8%	2.0	Y	Y			Y	Y		Y
Tawny owl *Strix aluco*	TO	Finland	[56]	38	350	83.3%	11.6		Y		Y		Y		
Western bluebird *Sialia mexicana*	WB	USA	[57, 58]	15	290	55.4%	2.1	Y	Y		Y	Y	Y		
Total					5,199	65.2%									

^1^The number of unique pairs for which both members were measured at least once.

^2^The proportion of pairs for which multiple morphological measurements were available for at least one member. ^3^The average number of male-measurement by female-measurement combinations that can be created per pair (e.g., male partner measured two times, female partner measured three times leads to 2 × 3 = 6 combinations).

Overall, the data include 32 population–trait combinations and 16,543 pair–trait combinations from a total of 5,199 pairs.

Given these selection criteria, we expect no bias with regard to assortative mating. We used these data in two ways: (1) to compare them with data from the literature search (see above) to assess the extent of search and reporting bias and (2) to quantify the extent to which correlations among pair members are affected by shared confounding effects (observer bias, temporal, and spatial autocorrelation). For the latter analyses, not all data sets contained all necessary information (e.g., observers who measured the pairs are unknown), but we used all available information.

#### Data handling

The previously unpublished data consist of two data sets. (1) All the pairs that have been identified across the nine studies for which both pair members have at least one morphological record (*n* = 6,309, including repeated records from different years). This data set also includes latitude and longitude of the nest site (Lambert azimuthal equal-area projection, units = meters) and year and, if available, the putative date of the first egg (S1 Data). (2) All available records of morphological traits (*n* = 41,896, which covered more than 95% of individuals included in [1], see Table 1). This data set also includes the location where the individual was caught, the date of catching, and the observer who measured the individual (S2 Data). We then combined the information from data tables (1) and (2) (S3 Data).

In most pairs (65.2%, see Table 1), one or both partners had been measured repeatedly for a given trait (regardless of whether they were paired at the time of measurement). For example, the female might have been weighed twice and the male three times. In this case, there are six combinations to align the measurements of the partners (2 x 3). The number of such combinations per pair (range 1 to 196) varied between studies (mean = 4.4, median = 2; Table 1) and allowed for a total of 72,739 combinations of male measurement by homologous female measurement.

Each of these combinations can be characterized by the circumstances of measurement (place, time, and observer) for each of the partners. We considered the pair members as measured at the “same site” (*n* = 26,542, 36.5%) if the Euclidean distance between their sites of capture (usually the nest) was less than 10 m or at “different sites” if they were caught more than 10 m apart (*n* = 44,112, 60.6%; the remaining 2.9% were cases of missing information). The 10-m threshold was chosen blind to the outcome of the analysis, but using other thresholds lead to similar results (S3 Fig). Likewise, combinations of measurements were defined as from the “same month” (*n* = 20,729, 28.5%) if obtained less than 30 days apart or as from “different months” (*n* = 51,995, 71.5%; *n* = 15 cases of missing data). The 30-day threshold was chosen blind to the outcome of the analysis, but using other thresholds lead to similar results (S3 Fig). Combinations of measurements were either from the “same observer” (*n* = 35,018, 48.1%) or from “different observers” (*n* = 24,771, 34.1%; 17.8% are missing data). Finally, for each of the 16,543 unique pair–trait combinations, we also selected the combination of measurements that was closest to the presumed date of pair formation (referred to as “nearest” to pairing). Because the date of pairing was unknown, we used the date of laying the first egg of their first recorded nest as a proxy (missing dates inferred from mean laying dates).

#### Estimating assortative mating

We estimated the strength of assortative mating by calculating Pearson’s correlation coefficients (r) and their 95% CI using six different approaches (models 1–6 below) that essentially differ in how the available morphological measurements are used.

The “random alignment model” (model 1)

For each combination of study and trait (*n* = 32), we first randomly sampled (1,000 times) from each pair one of the available male–female combinations of measurements and then calculated r and its 95% CI (averaged across the 1,000 replicates). We then summarized the 32 average correlation coefficients, weighed by sample size (*n* − 3)^0.5^, in which *n* is the number of pairs, using a mixed effect model with “Study” and “Trait” as random effects. This approach reflects the strength of assortment under “random” measuring conditions to the extent allowed by the data, i.e., given that 38% of the data were still from the same site, 29% from the same month, and 59% from the same observer.

The “average model” (model 2)

For each combination of study and trait (*n* = 32), we calculated r-values (and 95% CI) using mean trait values for each individual. This approach approximates the individuals’ average phenotype and is similar to the one used in quantitative genetics to estimate the underlying breeding value.

The “nearest model” (model 3)

In this model, we used the measurements taken closest in time to pair formation (see above) to calculate r-values (and their 95% CI) between pair members. This approach reflects the phenotypes around the time of pair formation, when mate choice can take place.

Model to reveal observer effect (model 4)

We first investigated with a set of simulations how estimates of “assortative mating” change with increasing simulated “true” levels of assortative mating and in relation to whether pairs were measured by the same or different observers (i.e., whether or not, for each pair, the same person measured both the male and the female; S1 Fig). We found that if the “true” level of assortative mating is close to zero, the correlation coefficients based on pairs measured by different observers were close to that “true” level. Thus, for each study–trait combination for which multiple observers had contributed data (*n* = 22 out of the 32 study–trait combinations, excluding Barn swallows and Western bluebirds), we calculated two r-values: one that included all pair combinations measured by the “same observer” (22 correlations, *n*_combinations_: range = 161–5,837, mean = 1,514) and one across all pair combinations measured by “different observers” (22 correlations, *n*_combinations_: range = 70–3,227, mean = 1,172, measured by on average 17 observers). These 44 correlation coefficients were summarized in a mixed model as described above: weighted by the number of pair combinations, with “Study,” “Trait,” and “Study-trait combination” as random effects and with observer category (same or different) as the fixed effect of interest.

Model to reveal temporal autocorrelation effect (model 5)

The model specifications are the same as in the previous model (4), but we contrasted r-values from pairs in which the members had been measured in the “same month” (32 correlations, n_combinations_: range = 58–3,064, mean = 648) versus in “different months” (31 correlations, n_combinations_: range = 223–6,204, mean = 1,677).

Model to reveal spatial autocorrelation effects (model 6)

The model specifications are the same as in the previous two models (4 and 5), but we contrasted r-values from pairs in which the members had been measured at the “same site” (32 correlations, n_combinations_: range = 210–2,773, mean = 829) versus at “different sites” (32 correlations, n_combinations_: range = 7–6,201, mean = 1,379).

### Experimental data on Zebra finches

We also assessed assortative mating for size experimentally, using captive populations of Zebra finches (*Taeniopygia guttata*). As a rule, in each experiment, all birds were measured by a single observer prior to their release into breeding aviaries. Measurements were taken in an order that was independent of allocation to aviaries. This excludes systematic observer error as well as spatial and temporal heterogeneity. To minimize the “scale-of-choice-effect” (when mate choice exists at a smaller scale than that of the investigator’s sampling, while the trait is heterogeneously distributed at the true scale-of-choice) [8, 15, 59], we analyzed the degree of assortative mating within aviaries, hence comprising only the birds that were available for pairing at the time of release. To avoid selective reporting, we summarize all available information from our laboratory (partly published in [25]), comprising five experiments that largely fulfill the above criteria (minor violations of criteria are described in the S1 Text; decisions about inclusion were made blind to the outcome of the analyses).

The five experiments involved both a domesticated population (experiments 1–3; [25]) and a recently wild-derived population (experiments 4–5 [24]; details are given in the S1 Text). In the domesticated population, we released six males and six females in each aviary (*n* = 6, 6, and 72 aviaries in experiments 1–3, respectively), while in the wild-derived population we used 20 males and 20 females per aviary (*n* = 4 and 2 aviaries in experiments 4 and 5, respectively). The formation of pair bonds was deduced from observations of pair-bonding behavior (allopreening, sitting in body contact, or visiting a nest box together). We only considered monogamous pair bonds (cases of polygyny and polyandry were excluded) but allowed sequential bonds (initial pairing followed by divorce and repairing; for details see [25]). In total, we recorded 510 pair bonds (*n* = 44, 35, 342, 58, and 31 in experiments 1–5, respectively). For the domesticated population, we measured body mass, tarsus length, and wing length; for the recently wild-derived population, we measured body mass and tarsus length. Thus, we had data for 13 experiment–trait combinations (3 x 3 plus 2 x 2). For each of these combinations, we calculated the strength of assortative mating (Pearson’s r) within each experimental aviary. To summarize the multiple estimates for the same trait from multiple aviaries within an experiment, we Fisher’s z-transformed the r-values and calculated a weighted average (weighted by [*n −* 3]^0.5^, in which *n* is the number of pairs, requiring a minimum of four pairs within an aviary). The 13 average estimates (based on 1,414 pair–trait combinations; morphological data missing for 27 pair–trait combinations) were back-transformed to correlation coefficients to allow comparison with all other results.

### Comparing the strength of assortative mating depending on the method

To compare the results across the different methods, we modeled the Pearson’s r estimates as a function of “data source” (fixed effect with four levels: “Web of Science search,” “Cited studies,” previously “Unpublished data,” and “Experimental data”). “Species” and “Study” were added as random effects to account for nonindependence of data. Moreover, different measures of size might yield different levels of assortment, so we fitted “Trait” as a third random effect—seven levels, reflecting the following categories: (1) body mass, (2) principal components of size, (3) bill measurements, (4) head measurements, (5) leg measurements, (6) tail measurements, and (7) wing measurements. Each Pearson’s r estimate was weighted by (*n −* 3)^0.5^, in which *n* is the total number of unique pairs contributing to the estimate. The analysis included 392 r-values from “Web of Science search” (based on 26,076 pairs), 57 r-values from “Cited studies” (based on 9,515 pairs), 32 r-values from the previously “Unpublished data” (based on 16,611 pairs), and 13 r-values from “Experimental data” on Zebra finches (based on 1,414 pairs). For the previously “Unpublished data,” we used estimates from the “nearest model 3” because this method is most frequently used in the published literature. Hence, differences between “Web of Science search,” “Cited studies,” and previously “Unpublished data” estimates should reflect search and reporting bias rather than methodological differences.

To examine the estimates from the literature (“Web of Science search” and “Cited studies”) for signs of publication bias, we plotted all 449 correlation coefficients over their sample size (number of pairs) and tested for asymmetry in “funnel plots” using the package “meta” in R 3.1.1 [60]. For comparisons and contrasts between published and previously unpublished data sets, the same plotting was done for previously unpublished data as well.

## Supporting information

S1 Data Previously unpublished data (nine long-term field studies). All the pairs that have been identified across the nine studies in which both pair members have at least one morphological record, including repeated records from different years. This data set also includes latitude and longitude of the nest site (Lambert azimuthal equal-area projection, units = meters) and year and, if available, the putative date of the first egg.
(XLSX)

S2 Data Previously unpublished data (nine long-term field studies). All available records of morphological traits which covered more than 95% of individuals included in S1 Data. This data set also includes the location where the individual was caught, the date of catching, and the observer who measured the individual.
(XLSX)

S3 Data Previously unpublished data (nine long-term field studies). Data in which we combined the information from S1 and S2 Data.
(XLSX)

S4 Data Published data (Fig 1A). Excel spreadsheet containing two separate sheets with data from literature: 1) data extracted from the publications and 2) the original references.
(XLSX)

S5 Data Data for Fig 1B. Strength of assortative mating for size as a function of data source.
(XLSX)

S6 Data Models for the previously unpublished data (Fig 1C). Excel spreadsheet contains six separate sheets; each sheet corresponds to a model to estimate the strength of assortative mating.
(XLSX)

S7 Data Data for Fig 2. Strength of assortative mating for body size in relation to sample size.
(XLSX)

S8 Data Experimental data on Zebra finches, including five experiments. (XLSX)

S1 Fig Simulated observer effects. We simulated 10 million pairs measured by 17 observers, which corresponds to the mean number of observers in the previously unpublished data of this study. Phenotypes of pair members (both sexes) were sampled from a normal distribution with mean = 70 mm (e.g., wing length) and a between-individual SD of 4 mm. Correlations of known magnitude between pair members were induced by splitting the between-individual variance into two components of varying size (a shared pair variance and a variance of individual deviation). Observer effects (a value of error for each observer) were sampled from a normal distribution with mean = 0 and SD = 1 mm. The *x*-axis shows the range of true levels of assortative mating (before adding observer error) that we modeled. The *y*-axis shows the difference between the observed correlation (after adding observer error) and the true correlation (positive values stand for overestimation, negative values for underestimation). The dark blue line (“same”) indicates the magnitude of overestimation when pair members are measured by the same observer, while the light blue line (“different”) shows that Pearson’s r is underestimated when all pair members are measured by different observers. The lines in between correspond to data sets that are composed by varying fractions of data stemming from those two conditions (80% “same” for our “nearest model;” 58.5% “same” for our “random model;” and 5.9% (= 1/17) “same” when observers are allocated randomly to measure individuals, purple line: “representative”). Note that the problem of overestimation is greater than the problem of underestimation, particularly when the true correlation is close to zero (as in our empirical data).
(TIF)

S2 Fig Assortative mating for eight morphological measures of size from previously “Unpublished data” (nine field studies, species name abbreviations in Table 1; details in S2, S3 and S4 Tables). A–C. Dots represent estimated assortative mating, bars the 95% CI, and color body size trait for “random alignment model” (A), “average model” (B), and “nearest model” (C). The estimates of Semipalmated sandpipers’ wing and Tawny owl’s mass are the highest (significant positive assortment) across these three models. Note that the pair of measurements for each pair and trait were randomly sampled from the available male–female combinations of measurements (A), were averages of all available combinations of measures of pair members (B), and were the measures of pair members that were taken closest to the presumed time of pair formation (C).
(TIF)

S3 Fig Change in estimates in relation to different thresholds for defining “same” and “different” for variation in time and space. Left panels: dots represent mean Pearson’s correlation coefficients (r), bars the 95% CI based on the same time (top) and space (bottom) models as in Fig 1 (S5 Table). Right panels: distribution of sample sizes (number of pairs) for each threshold value. Note that differences between the conditions “same” and “different” are stable across a wide range of thresholds, except when sample sizes for one category become small.
(TIF)

S1 Table Summary of the strength of assortative mating from literature data across eight types of traits. The mixed-effect model includes 825 estimates from “Web of Science search” and “Cited studies.” Pearson’s correlation coefficients of assortment (weighed by sample size (*n* − 3)^0.5^, *n* = number of pairs) are modeled as the response variable. *P* values were calculated from t-values with infinite df. The overall intercept was removed to directly show the average Pearson’s correlation for each trait category (fixed effect with eight levels). The random effect estimates show the proportion of variation explained (repeatability).
(DOCX)

S2 Table Assortative mating estimates from the “random alignment model.” For each study–trait combination, the average Pearson’s r and the average boundaries of the 95% CI (from 1,000 simulations) and the number of unique pairs are indicated. Asterisks mark significant (*P* < 0.05) correlations.
(DOCX)

S3 Table Assortative mating estimates from the “average model.” For each study–trait combination, the Pearson’s r, the boundaries of the 95% CI, and the number of unique pairs are indicated. Asterisks mark significant (*P* < 0.05) correlations. Note that 27 out of 32 correlations are higher than those from S2 Table.
(DOCX)

S4 Table Assortative mating estimates from the “nearest model” (model 3). For each study–trait combination, the Pearson’s r, the boundaries of the 95% CI, and the number of unique pairs are indicated. Asterisks mark significant (*P* < 0.05) correlations. Note that 27 out of 32 correlations are higher than those from S2 Table.
(DOCX)

S5 Table Assortative mating estimates with and without observer bias, temporal and spatial autocorrelation. The fixed-effect level “Same” refers to measurements from the same observer, from the same month, and from the same site (while “Different” refers to measurements from different observers, measurements taken more than 30 days apart, or measurements taken more than 10 m apart. The overall intercept was removed to directly show the average degree of assortative mating and 95% CI for each of the two levels of the fixed effect and its significance in terms of t-values and *P* values (calculated with infinite df). The random effects estimates show the proportion of variance explained (repeatability).
(DOCX)

S6 Table Data summary for experimental studies on captive Zebra finches. Here, each correlation estimate r is the weighted (by [*n–* 3]^0.5^, with *n* = number of pairs) average of correlation coefficients calculated within experimental aviaries. Experiments are numbered as in the Supplementary Methods section. Tarsus length from experiments 4 and 5 (marked with asterisks) were measured after releasing the birds into the aviaries.
(DOCX)

S7 Table The degree of assortative mating as a function of data source. The overall intercept was removed to directly show the average degree of assortative mating and 95% CI for each of the four levels of the fixed effect and its significance in terms of t-values and *P* values (calculated with infinite df). The random effects show the proportion of variance explained (repeatability). Pearson’s r estimates for previously “Unpublished data” field studies were taken from the “nearest model” (see model 3 in methods section). *N* = 494 Pearson’s r estimates.
(DOCX)

S1 Text Description of nine long-term field studies and experimental Zebra finch data. (DOCX)

S1 PRISMA checklist The process of literature investigation. (DOCX)
