# Supplementary figures and images for: Scrutinizing assortative mating in birds
Source: PLoS Biol. 2019 Feb 21;17(2):e3000156. doi: 10.1371/journal.pbio.3000156 (PMC6400405; doi:10.1371/journal.pbio.3000156)

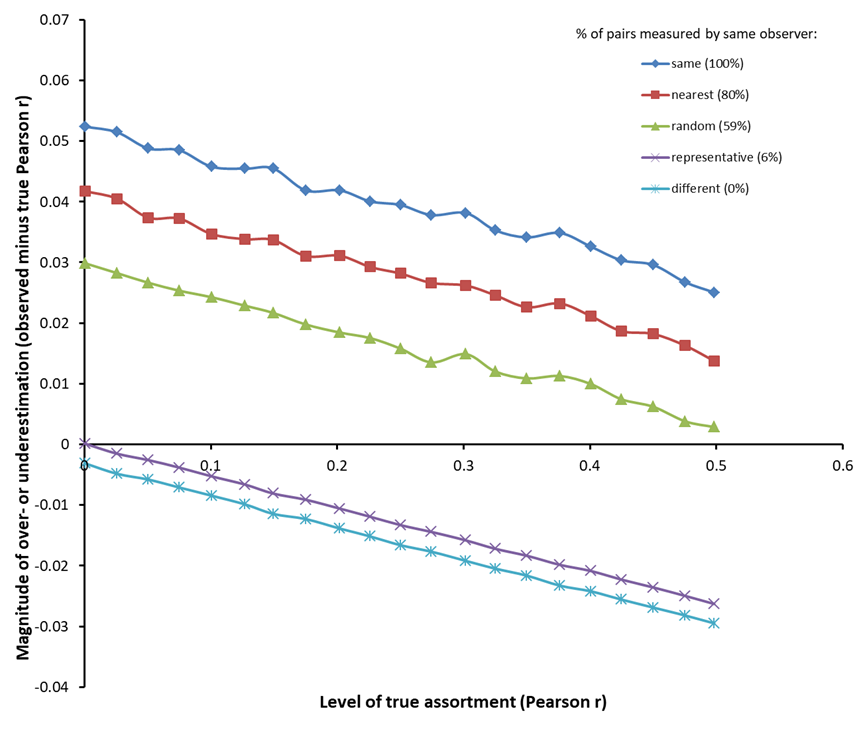

Supplement: S1 Fig — We simulated 10 million pairs measured by 17 observers, which corresponds to the mean number of observers in the previously unpublished data of this study. Phenotypes of pair members (both sexes) were sampled from a normal distribution with mean = 70 mm (e.g., wing length) and a between-individual SD of 4 mm. Correlations of known magnitude between pair members were induced by splitting the between-individual variance into two components of varying size (a shared pair variance and a variance of individual deviation). Observer effects (a value of error for each observer) were sampled from a normal distribution with mean = 0 and SD = 1 mm. The x-axis shows the range of true levels of assortative mating (before adding observer error) that we modeled. The y-axis shows the difference between the observed correlation (after adding observer error) and the true correlation (positive values stand for overestimation, negative values for underestimation). The dark blue line (“same”) indicates the magnitude of overestimation when pair members are measured by the same observer, while the light blue line (“different”) shows that Pearson’s r is underestimated when all pair members are measured by different observers. The lines in between correspond to data sets that are composed by varying fractions of data stemming from those two conditions (80% “same” for our “nearest model;” 58.5% “same” for our “random model;” and 5.9% (= 1/17) “same” when observers are allocated randomly to measure individuals, purple line: “representative”). Note that the problem of overestimation is greater than the problem of underestimation, particularly when the true correlation is close to zero (as in our empirical data). (TIF) [file pbio.3000156.s009.tif]

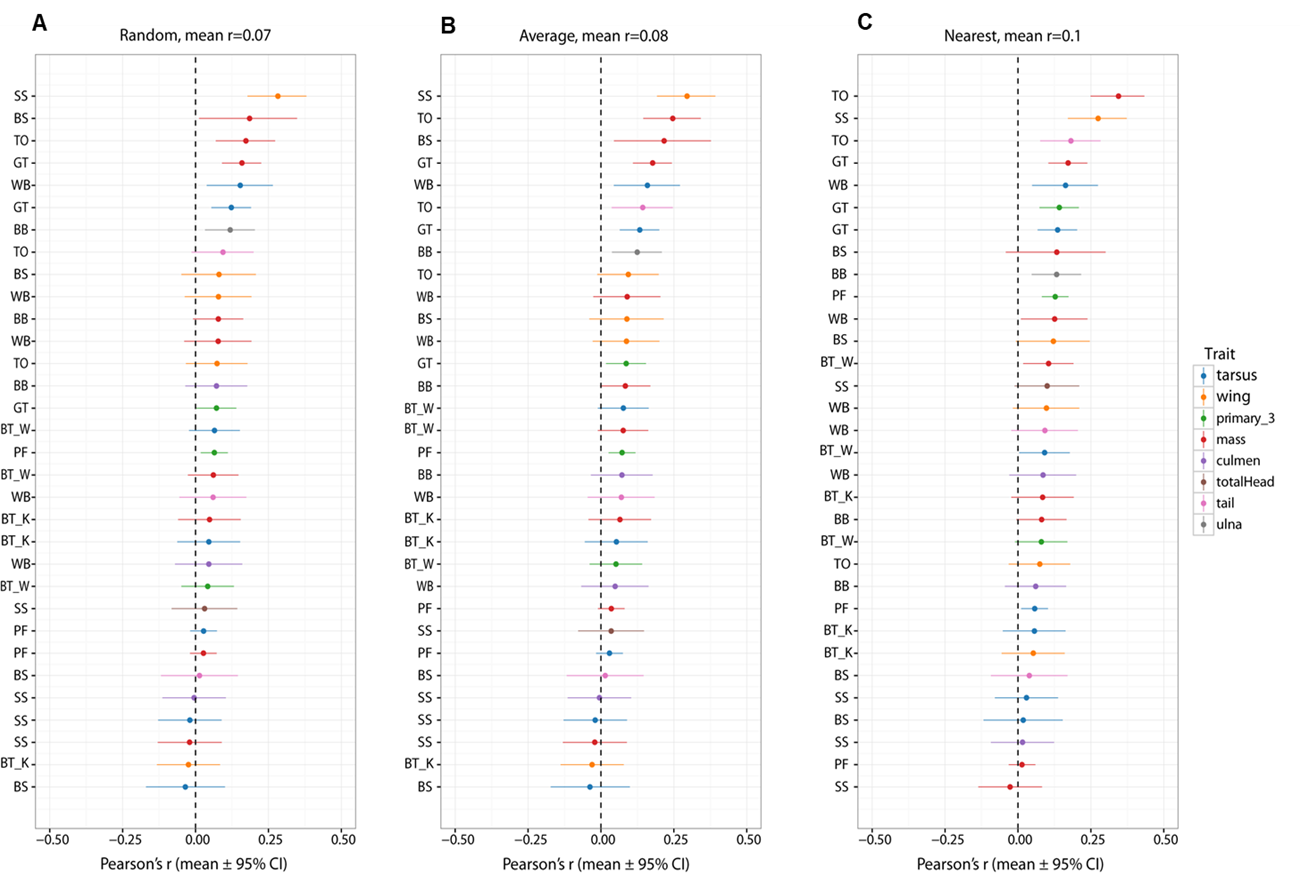

Supplement: S2 Fig — A–C. Dots represent estimated assortative mating, bars the 95% CI, and color body size trait for “random alignment model” (A), “average model” (B), and “nearest model” (C). The estimates of Semipalmated sandpipers’ wing and Tawny owl’s mass are the highest (significant positive assortment) across these three models. Note that the pair of measurements for each pair and trait were randomly sampled from the available male–female combinations of measurements (A), were averages of all available combinations of measures of pair members (B), and were the measures of pair members that were taken closest to the presumed time of pair formation (C). (TIF) [file pbio.3000156.s010.tif]

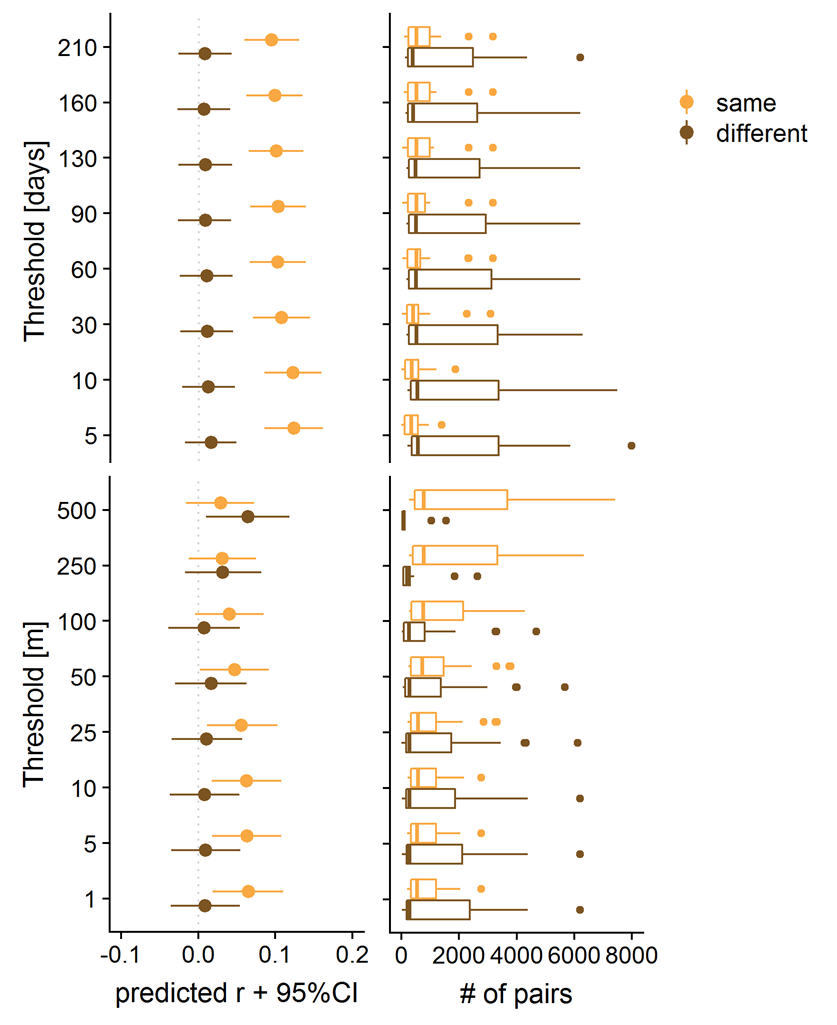

Supplement: S3 Fig — Left panels: dots represent mean Pearson’s correlation coefficients (r), bars the 95% CI based on the same time (top) and space (bottom) models as in Fig 1 (S5 Table). Right panels: distribution of sample sizes (number of pairs) for each threshold value. Note that differences between the conditions “same” and “different” are stable across a wide range of thresholds, except when sample sizes for one category become small. (TIF) [file pbio.3000156.s011.tif]

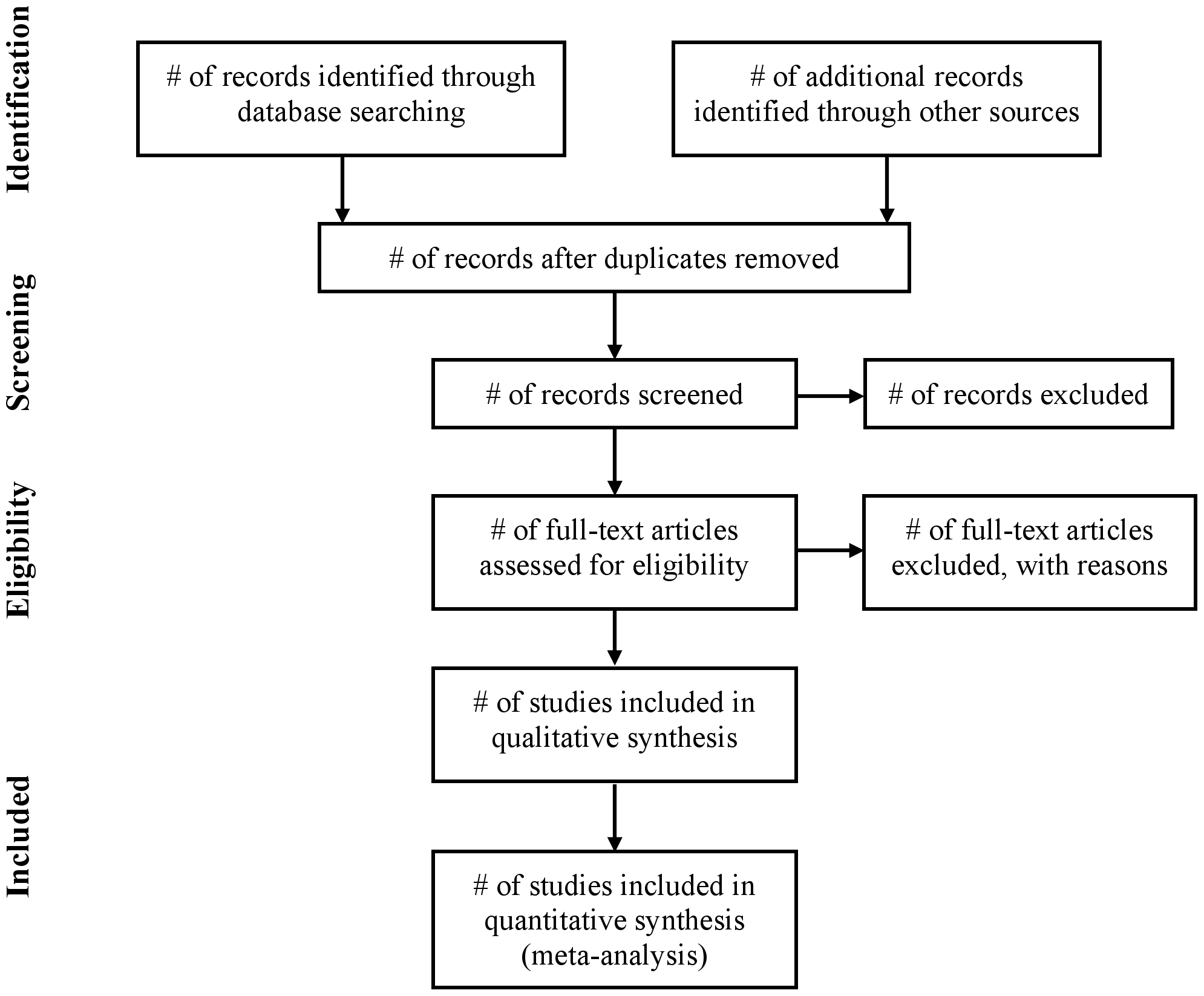


**71**

**12**

**569**

**40**

**529**

**330**

**199**

**199**

**510**406

Supplement: S1 PRISMA checklist — (DOCX) [file pbio.3000156.s020.docx]
