# Supplementary material for: Scrutinizing assortative mating in birds
Source: PLoS Biol. 2019 Feb 21;17(2):e3000156. doi: 10.1371/journal.pbio.3000156 (PMC6400405; doi:10.1371/journal.pbio.3000156)
Supplement: S1 Table — The mixed-effect model includes 825 estimates from “Web of Science search” and “Cited studies.” Pearson’s correlation coefficients of assortment (weighed by sample size (n − 3)0.5, n = number of pairs) are modeled as the response variable. P values were calculated from t-values with infinite df. The overall intercept was removed to directly show the average Pearson’s correlation for each trait category (fixed effect with eight levels). The random effect estimates show the proportion of variation explained (repeatability). (DOCX) [file pbio.3000156.s012.docx]

S1 Table.

|  |  | |  | 95% CI | |  |  |
| --- | --- | --- | --- | --- | --- | --- | --- |
|  | # of r | | Estimate | Lower | Upper | t | P |
| random effects: |  |  | |  |  |  |  |
| Study ID | 199 | 9% | |  |  |  |  |
| Species ID | 139 | 6% | |  |  |  |  |
| Residual |  | 85% | |  |  |  |  |
|  |  |  | |  |  |  |  |
| fixed effects: |  |  | |  |  |  |  |
| Age | 46 | 0.434 | | 0.364 | 0.504 | 12.12 | <0.0001 |
| Behaviour | 42 | 0.313 | | 0.228 | 0.398 | 7.24 | <0.0001 |
| Body condition | 34 | 0.215 | | 0.139 | 0.291 | 5.56 | <0.0001 |
| Body size | 449 | 0.194 | | 0.151 | 0.237 | 8.88 | <0.0001 |
| Heterozygosity | 21 | 0.156 | | 0.041 | 0.271 | 2.65 | 0.008 |
| Others | 14 | 0.310 | | 0.185 | 0.435 | 4.86 | <0.0001 |
| Physiology | 20 | 0.335 | | 0.224 | 0.446 | 5.91 | <0.0001 |
| Plumage | 199 | 0.269 | | 0.218 | 0.320 | 10.39 | <0.0001 |
