# Supplementary material for: Scrutinizing assortative mating in birds
Source: PLoS Biol. 2019 Feb 21;17(2):e3000156. doi: 10.1371/journal.pbio.3000156 (PMC6400405; doi:10.1371/journal.pbio.3000156)
Supplement: S4 Table — For each study–trait combination, the Pearson’s r, the boundaries of the 95% CI, and the number of unique pairs are indicated. Asterisks mark significant (P < 0.05) correlations. Note that 27 out of 32 correlations are higher than those from S2 Table. (DOCX) [file pbio.3000156.s015.docx]

S4 Table.

| Study species | Traits | r | 95% CI low | 95% CI up | N pairs |
| --- | --- | --- | --- | --- | --- |
| Blue-footed booby | culmen | 0.06 | -0.04 | 0.17 | 346 |
| Blue-footed booby | mass | 0.08 | -0.01 | 0.17 | 515 |
| Blue-footed booby | ulna | 0.13^*^ | 0.05 | 0.22 | 517 |
| Barn swallow | tarsus | 0.02 | -0.12 | 0.15 | 209 |
| Barn swallow | tail | 0.04 | -0.09 | 0.17 | 222 |
| Barn swallow | wing | 0.12 | -0.01 | 0.25 | 233 |
| Barn swallow | mass | 0.13 | -0.04 | 0.30 | 127 |
| Great tit | primary 3 | 0.14^*^ | 0.07 | 0.21 | 811 |
| Great tit | tarsus | 0.14^*^ | 0.07 | 0.20 | 809 |
| Great tit | mass | 0.17^*^ | 0.10 | 0.24 | 809 |
| Blue tit_K | wing | 0.05 | -0.06 | 0.16 | 328 |
| Blue tit_K | tarsus | 0.06 | -0.05 | 0.16 | 330 |
| Blue tit_K | mass | 0.08 | -0.02 | 0.19 | 331 |
| Pied flycatcher | tarsus | 0.06^*^ | 0.01 | 0.10 | 1824 |
| Pied flycatcher | mass | 0.01 | -0.03 | 0.06 | 1838 |
| Pied flycatcher | primary 3 | 0.13^*^ | 0.08 | 0.17 | 1795 |
| Semipalmated sandpiper | mass | -0.03 | -0.14 | 0.08 | 322 |
| Semipalmated sandpiper | tarsus | 0.03 | -0.08 | 0.14 | 329 |
| Semipalmated sandpiper | culmen | 0.02 | -0.09 | 0.12 | 329 |
| Semipalmated sandpiper | totalHead | 0.10 | -0.01 | 0.21 | 306 |
| Semipalmated sandpiper | wing | 0.28^*^ | 0.17 | 0.37 | 324 |
| Tawny owl | wing | 0.07 | -0.03 | 0.18 | 343 |
| Tawny owl | tail | 0.18^*^ | 0.08 | 0.28 | 337 |
| Tawny owl | mass | 0.34^*^ | 0.25 | 0.43 | 351 |
| Western bluebird | culmen | 0.09 | -0.03 | 0.20 | 289 |
| Western bluebird | tail | 0.09 | -0.02 | 0.21 | 290 |
| Western bluebird | wing | 0.10 | -0.02 | 0.21 | 291 |
| Western bluebird | mass | 0.13^*^ | 0.01 | 0.24 | 287 |
| Western bluebird | tarsus | 0.16^*^ | 0.05 | 0.27 | 286 |
| Blue tit_W | primary 3 | 0.08 | -0.01 | 0.17 | 471 |
| Blue tit_W | tarsus | 0.09^*^ | 0.00 | 0.18 | 503 |
| Blue tit_W | mass | 0.10^*^ | 0.02 | 0.19 | 509 |
