# Supplementary material for: Scrutinizing assortative mating in birds
Source: PLoS Biol. 2019 Feb 21;17(2):e3000156. doi: 10.1371/journal.pbio.3000156 (PMC6400405; doi:10.1371/journal.pbio.3000156)
Supplement: S5 Table — The fixed-effect level “Same” refers to measurements from the same observer, from the same month, and from the same site (while “Different” refers to measurements from different observers, measurements taken more than 30 days apart, or measurements taken more than 10 m apart. The overall intercept was removed to directly show the average degree of assortative mating and 95% CI for each of the two levels of the fixed effect and its significance in terms of t-values and P values (calculated with infinite df). The random effects estimates show the proportion of variance explained (repeatability). (DOCX) [file pbio.3000156.s016.docx]

S5 Table.

|  |  |  |  | 95% CI | |  | |  |
| --- | --- | --- | --- | --- | --- | --- | --- | --- |
| Model | Effect type | Effect | Estimate | Lower | Upper | | t | p |
| Observers | Random | Trait (n =8) | 0% |  |  | |  |  |
|  | (variance) | Study (n = 7) | 1.3% |  |  | |  |  |
|  |  | Trait × Study (n = 22) | 0% |  |  | |  |  |
|  |  | Residual | 98.7% |  |  | |  |  |
|  | Fixed | Same | 0.075 | 0.036 | 0.114 | | 3.45 | < 0.0001 |
|  |  | Different | 0.023 | -0.016 | 0.062 | | 1.01 | 0.31 |
|  |  |  |  |  |  | |  |  |
| Month | Random | Trait (n =8) | 0% |  |  | |  |  |
|  | (variance) | Study (n = 9) | 0.5% |  |  | |  |  |
|  |  | Trait × Study (n =32) | 0 |  |  | |  |  |
|  |  | Residual | 99.5% |  |  | |  |  |
|  | Fixed | Same | 0.110 | 0.071 | 0.149 | | 6.92 | < 0.0001 |
|  |  | Different (> 30 days) | 0.014 | -0.025 | 0.053 | | 0.97 | 0.33 |
|  |  |  |  |  |  | |  |  |
| Site | Random | Trait (n = 8) | 0% |  |  |  | |  |
|  | (variance) | Study (n = 9) | 0.6% |  |  |  | |  |
|  |  | Trait × Study (n =32) | 0.6% |  |  |  | |  |
|  |  | Residual | 98.8% |  |  |  | |  |
|  | Fixed | Same | 0.073 | 0.053 | 0.093 | 5.17 | | < 0.0001 |
|  |  | Different (>10m) | 0.017 | -0.003 | 0.037 | 1.28 | | 0.2 |
|  |  |  |  |  |  |  | |  |
