# Supplementary material for: Scrutinizing assortative mating in birds
Source: PLoS Biol. 2019 Feb 21;17(2):e3000156. doi: 10.1371/journal.pbio.3000156 (PMC6400405; doi:10.1371/journal.pbio.3000156)
Supplement: S6 Table — Here, each correlation estimate r is the weighted (by [n– 3]0.5, with n = number of pairs) average of correlation coefficients calculated within experimental aviaries. Experiments are numbered as in the Supplementary Methods section. Tarsus length from experiments 4 and 5 (marked with asterisks) were measured after releasing the birds into the aviaries. (DOCX) [file pbio.3000156.s017.docx]

S6 Table.

| Experiment | Population | Trait | n pairs | n aviaries | r |
| --- | --- | --- | --- | --- | --- |
| 1 | domesticated | mass | 44 | 6 | 0.01 |
| 1 | domesticated | tarsus | 44 | 6 | 0.25 |
| 1 | domesticated | wing | 44 | 6 | -0.26 |
| 2 | domesticated | mass | 35 | 6 | -0.45 |
| 2 | domesticated | tarsus | 35 | 6 | 0.20 |
| 2 | domesticated | wing | 35 | 6 | 0.07 |
| 3 | domesticated | tarsus | 331 | 67 | -0.25 |
| 3 | domesticated | mass | 336 | 68 | -0.10 |
| 3 | domesticated | wing | 336 | 68 | -0.12 |
| 4 | wild-derived | mass | 31 | 2 | 0.30 |
| 4 | wild-derived | tarsus* | 29 | 2 | 0.38 |
| 5 | wild-derived | mass | 58 | 4 | 0.27 |
| 5 | wild-derived | tarsus* | 56 | 4 | 0.21 |
