# Supplementary material for: Scrutinizing assortative mating in birds
Source: PLoS Biol. 2019 Feb 21;17(2):e3000156. doi: 10.1371/journal.pbio.3000156 (PMC6400405; doi:10.1371/journal.pbio.3000156)
Supplement: S7 Table — The overall intercept was removed to directly show the average degree of assortative mating and 95% CI for each of the four levels of the fixed effect and its significance in terms of t-values and P values (calculated with infinite df). The random effects show the proportion of variance explained (repeatability). Pearson’s r estimates for previously “Unpublished data” field studies were taken from the “nearest model” (see model 3 in methods section). N = 494 Pearson’s r estimates. (DOCX) [file pbio.3000156.s018.docx]

S7 Table.

|  |  |  | 95% CI | | |  |  |
| --- | --- | --- | --- | --- | --- | --- | --- |
|  | # of r estimates | Estimates | Lower | | Upper | t | p |
| Random effects: |  |  |  |  | |  |  |
| Study | 106 | 7% |  |  | |  |  |
| Species | 89 | 0% |  |  | |  |  |
| Trait-type | 7 | 0% |  |  | |  |  |
| Residual |  | 93% |  |  | |  |  |
|  |  |  |  |  | |  |  |
| Fixed effects: |  |  |  |  | |  |  |
| Web of Science search | 392 | 0.184 | 0.147 | 0.221 | | 9.91 | <0.0001 |
| Cited studies | 57 | 0.140 | 0.060 | 0.220 | | 3.47 | 0.001 |
| Unpublished data | 32 | 0.107 | 0.017 | 0.197 | | 2.31 | 0.020 |
| Experimental data | 13 | -0.004 | -0.267 | 0.259 | | -0.02 | 0.976 |
