# Supplementary material for: Scrutinizing assortative mating in birds
Source: PLoS Biol. 2019 Feb 21;17(2):e3000156. doi: 10.1371/journal.pbio.3000156 (PMC6400405; doi:10.1371/journal.pbio.3000156)
Supplement: S1 Text — (DOCX) [file pbio.3000156.s019.docx]

**Supplementary Materials**

**Supplementary Methods**

***Description of long-term field studies (‘Unpublished data’)***

***(1) Barn swallows***

Barn swallows were studied in four separate breeding colonies in the Trebon area, South Bohemia, Czech Republic, between 2010 and 2015 (six breeding seasons). All birds were captured during the early breeding season and wing length, tarsus length and body mass measured. Right and left tail streamer lengths were measured to the nearest mm, and we use the average of the two measures as tail length. Each individual received an aluminium ring (National Museum Prague) and a unique combination of plastic colour rings (AVINET) before release. Phenotypic (morphological) measurements were taken early in the season, while members of social pairs were identified later in the season by the colour band combination of individuals that incubated or provisioned offspring at active nests. Nests were checked daily to determine the onset of egg laying. In the analysis, we only included first breeding attempts of each social pair in each year. For further details see Petrzelkova et al. (2015) and Wilkins et al. (2016).

***(2) Blue-footed boobies***

Blue-footed boobies were studied at the Isla Isabel colony off the Pacific coast of Mexico. Since 1988, reproduction has been monitored each year by marking nests, recording nest contents, and banding nestlings between February and July, and >90% of the breeders in the study area were banded with a unique number(Drummond et al. 2003). Between 2010 and 2013, culmen, ulna, and body mass were measured for a total of 551 pairs (510 unique pairs). This sample comprised two subsamples: (1) 170 pairs measured between December and March before egg laying; these pairs were defined based on behaviours including mutual courting, allopreening, and joint territory defence over 4-5 days of behavioural observations prior to capture; (2) 381 pairs measured between February and April 2011 when their broods were 10-40 days old. For further details see Kiere et al. (2016).

***(3) Blue tits: study site Kolbeterberg***

A population of blue tits was studied in a 35 ha plot of mixed deciduous woodland in Vienna, Austria (48°139 N, 16°209 E). The forest is dominated by oak (*Quercus robur*), beech (*Fagus sylvatica*) and ash (*Fraxinus excelsior*) and contained maximally 220 nest-boxes. We captured blue tits in their nestbox, either in winter while they were roosting or in late spring during nestling feeding. Unbanded birds were marked with a unique combination of plastic colour bands and a numbered metal ring. At capture, we measured tarsus and wing length with a calliper to the nearest 0.05 mm, and body mass with an electronic balance to the nearest 0.1 g. For more details see (Delhey et al. 2003; Foerster et al. 2003).

***(4) Blue tits at Westerholz***

The project is part of a long-term study on the breeding biology of blue tits, conducted in a mixed deciduous/coniferous woodland (‘Westerholz’, 48°08´26´´, N 10°53´29´´E) near Landsberg am Lech, southern Germany. The study area is an unmanaged part of the forest (‘Reiherschlag’, ca. 40 ha), which is dominated by mature oak trees and contains 277 nestboxes (since 2007) with 60-100 breeding attempts of blue tits each year. All breeding pairs were captured inside the nestbox, either in the winter preceding the breeding season (roosting), or during the breeding season (when adults fed 8-10-day-old nestlings, using an automated nestbox trap). We marked them with a unique combination of colour bands, took a small blood sample from the brachial vein (approximately 50 ml) for later parentage analysis, and measured tarsus, wing length and body mass. For more details see (Schlicht et al. 2012).

***(5) Great tits***

The studied population of great tits breeding in nest boxes is in Southern Germany (Bavarian Landkreis Starnberg; 47°58´N, 11°14´ E). The nest boxes were located in 12 plots established in 2009 with each plot approximately 9 hectares in size and consisting of a regular grid of 50 nest boxes with 50 m between adjacent boxes. Nest boxes were checked twice per week from April onward to determine lay date (back-calculated assuming that one egg was laid per day), onset of incubation and clutch size. Nestlings were blood sampled and marked with an aluminium ring when they were 6 days old. Parents were caught with a spring trap in the nest box the next day, measured, bled, and marked with a unique combination of rings if not ringed previously. For more details see (Araya-Ajoy et al. 2016).

***(6) Pied flycatchers***

Since 2007, breeding pairs of pied flycatchers (ca. 300) in Drenthe (NL, 52°49'N, 6°22'E) in ca. 1100 nest boxes distributed across 12 plots, 9 with 100 and 3 with ca. 50 nest boxes each. Pairs are defined as a male and a female that were caught during nestling feeding in a nest box (for over 90% of all nests the female identity was known and male identity was known for ca 85%). Polygyny is rather rare in this population (<4% in most years). We measured tarsus length (to the nearest 0.1 mm), the length of the third primary (from outside, to the nearest 0.5 mm) and body weight (to the nearest 0.1 g) of all birds upon capture. Several observers were measuring the birds during each year, and it was mostly the same observer measuring the male and female of a pair. Females were also caught (if possible) during incubation (around day 7 after clutch completion) and at this moment the females are considerably heavier than during nestling feeding. We did not always aim catching females again during nestling feeding if we knew their identity. For more details see (Both et al. 2017).

***(7) Semipalmated sandpipers***

A population of Semipalmated sandpipers was studied near Barrow, Alaska (71. 32° N, 156.65°W). Breeding adults were marked with an aluminium US Geological Survey band, a unique combination of 4 color bands, and a green flag with embedded glass passive–integrated tag (Biomark: 9.0 mm × 2.1 mm, 0.087 g, 134.2 kHz, ISO FDXB, http://www.biomark.com/). We took a small (ca. 50 μl) blood sample from the brachial vein for molecular sexing, weighed each bird (to the nearest 0.1 g) using a digital balance, and measured tarsus, culmen, and total head (to the nearest 0.1 mm) with callipers and wing length (to the nearest 0.5 mm) with a ruler. For more details see (Bulla et al. 2014).

***(8) Tawny owls***

Tawny owls were studied in a nest box equipped study area of ca. 250 km^2^ in southern Finland (60° 15’ N, 24° 15’ E) between 1978 and 2015. Throughout the study period nearly all pairs nested in nest boxes, which were provided in high abundance. Each year starting in mid-April, all boxes and other possible breeding sites were checked. Practically all females and males were trapped when the offspring were 1–2 weeks old. Brooding females were taken from their nest boxes in the evening by netting them at the opening of the nest box. After handling, the female was put back into the nest box and a swing-door trap for the male was mounted in front of it and left over night. In the following morning, traps were checked and the males were handled. During handling the parental birds were ringed (if unbanded) and their wing length and tail length were measured with a ruler and body mass was measured with a spring scale.

In this data set the definition of a pair is when both the male and the female has been caught and identified in the same breeding occasion. Tawny owls breed only once during a breeding season and do not re-nest if the breeding fails or the brood is depredated. The frequency of extra-pair young is low in tawny owls and estimated to 2.7 % in Saladin et al. (2007). More information on the study population and morphological traits were provided in (Karell et al. 2009; Brommer et al. 2015).

***(9) Western Bluebirds***

Data of Western bluebirds were collected over 15 breeding seasons (2001–2015) from a nest-box population of Western bluebirds in western Montana, USA (see Duckworth, 2006 for study site details). GPS coordinates for all nest boxes were recorded each year. Each year, nest boxes were visited at least twice weekly during the breeding season (April–August) to monitor nest progress, to determine the affiliation of breeding pairs with specific boxes, and to band offspring and adults. Adults were captured at each site using traps baited with mealworms to mark them with a unique colour band combination, and take standard morphological measurements, including body mass and length of the tarsus, tail, wing, and bill (for details on morphological variation see Duckworth and Semenov 2017). Individuals were identified as a breeding pair if they were observed together defending a territory and nest box and jointly participating in breeding activities (courtship feeding of female by male, male feeding female on nest, both parents feeding nestlings).

***Description of ‘Experimental data’***

***Morphological measurements of zebra finches***

All birds of the domesticated population (experiments 1-3 below) were measured by the same observer (W.F.) for body mass (to the nearest 0.1g) using electronic scales, for wing length (to the nearest 0.5mm) using a wing ruler, and for tarsus length (to the nearest 0.1mm) using a wing ruler, when they reached 100-120 days of age (prior to release into the experimental aviaries). All birds of the wild-derived population (experiments 4-5 below) were measured by Malika Ihle for body mass (to the nearest 0.1g) using electronic scales on the day of their release into the experimental aviaries (when reaching 45 days of age). Measurements of their tarsus length (to the nearest 0.1mm) using a wing ruler were all taken by Ulrich Knief (between 25-04-2012 and 04-05-2012) after the birds had formed pair bonds (when birds were 284 ± 46 days old, range 190 – 378 days). Note that the latter tarsus measurements violate the criterion of measuring before pair formation (hence the marking by asterisks in Table S6), yet we assume that tarsi are fully grown by 45 days of age and do not change thereafter.

***Observations of pair bonds in 5 experimental studies***

*(1) Domesticated population: inbreeding avoidance study 2007*

This experiment was designed to test whether cross-fostered zebra finches avoid pairing with unfamiliar genetic full-sibs (following up on Schielzeth et al. 2008). The studied domesticated population was kept at the Max Planck Institute for Ornithology in Seewiesen, Germany since 2004 (population # 18 in Forstmeier et al. 2007). Housing conditions, diet and aviary specifications for breeding have been described in detail in the Supplementary File to Wang et al. (2017). In this study, we used 36 males and 36 females that originated from 12 families (always 3 sons and 3 daughters that were all unfamiliar from each family). We used 6 experimental aviaries, each equipped with 6 nest boxes, and in each we released the members of two families (6 males and 6 females) to observe to which extent pair bonds form within and between families. The experiment lasted for 12 weeks (11-09-2007 to 03-12-2007). All birds were colour-banded for individual recognition (like in all following experiments). Observations of pair bonding behaviours (allopreening, sitting in body contact, and visiting a nest-box together) were carried out at least once per day, but around 6-8 times a day at the beginning of the experiment. We defined the start of one pair bond as the time when the female did not show any pair bonding behaviour anymore with another male. The end of a pair bond was defined by either the first observation of another exclusive pair bond (if applicable) or the last observation of pair bonding behaviour (if the pair bond did not seem to last until the end of the experiment). Some individuals engaged in multiple pair bonds, either sequentially (considered as monogamous) or simultaneously (polygamous). For this present analysis we only included monogamous pairs bonds that had been observed (n = 44).

*(2) Domesticated population: inbreeding depression study 2009*

This experiment was similar to the previous one, but it comprised the inbred and outbred offspring that had been produced during the previous experiment. Each of the 6 aviaries again received 6 males and 6 females (half inbred (F = 0.25), and half outbred (F = 0)) that were all unfamiliar. The experiment lasted 16 weeks (07-04-2009 to 28-07-2009). Following daily observations, 35 monogamous pair bonds were formed.

*(3) Domesticated population: selection lines 2014/15*

The details of this experiment have been described in Wang et al. (2017). Briefly, the birds are from the same captive population as described above. In 2009 we initiated the breeding of lines that were selected for high versus low breeding values for male courtship rate (two high lines, two unselected control lines, two low lines; see Mathot et al. (2013)). The third generation of these six lines consisted of a total of 343 females and 338 males. A subset of 219 females and 217 males (about equally representing the six lines) were randomly divided into 4 cohorts that were tested sequentially due to the limited number of aviaries (n = 9) available. Each cohort went through two rounds of breeding, in each of which they encountered a different set of potential partners over a 7 week period. During each breeding round of a cohort, we carried out daily observations as described above. Observations lasted approximately 30 min (total across the nine aviaries) and were carried out approximately 120 times per breeding round. Across the four cohorts and the two breeding rounds we identified a total of 423 pair bonds within the 72 aviaries. Of these, 342 bonds were classified as monogamous (see Wang et al. 2017) and included into this study.

*(4) Wild-derived population: compatibility study 2012*

This wild-derived population (described as population # 4 in Forstmeier et al. 2007) was derived from wild-caught birds from northern Victoria about 12-15 generations ago. In 1992, 12 males and 12 females had been exported to Bielefeld, Germany, and bred there. In 2009, 109 individuals were transferred from Bielefeld to Seewiesen, where the population has been maintained since. All birds of the experiment hatched in the summer of 2011 in large semi-outdoor aviaries. Shortly after independence (when birds were 45 days old), they were put into 8 mixed-sex peer-groups of 10 males and 10 females. When birds reached sexual maturity (100 days old) they were colour-banded individually, and peer-groups were joined two by two (yielding four groups, each allowing for 20 possible pairs to form). Following observations for pair bond identification (as described above), 58 pairs were identified during the winter of 2011/2012 and included into this study. For more details see Ihle et al. (2015).

*(5) Wild-derived population: inbreeding depression study 2012*

This experiment is identical to the previous one (experiment 4), yet it comprised a balanced mix of inbred (F = 0.25) and outbred (F = 0) offspring (like in experiment 2). When reaching 45 days of age, offspring went into four mixed-sex peer-groups (each group including five outbred males, five outbred females, five inbred males and five inbred females). When reaching about 100 days of age, the peer-groups were joined two by two in two different aviaries for the whole winter. Following observations as described above we identified 31 monogamous pairs that were included in this study.

Reference of Supplement

1 Petrzelkova, A. *et al.* Brood parasitism and quasi-parasitism in the European barn swallow Hirundo rustica rustica. *Behav Ecol Sociobiol* **69**, 1405-1414, doi:10.1007/s00265-015-1953-6 (2015).

2 Wilkins, M. R. *et al.* Phenotypic differentiation is associated with divergent sexual selection among closely related barn swallow populations. *J Evolution Biol* **29**, 2410-2421, doi:10.1111/jeb.12965 (2016).

3 Drummond, H., Torres, R. & Krishnan, V. V. Buffered development: Resilience after aggressive subordination in infancy. *Am Nat* **161**, 794-807, doi:Doi 10.1086/375170 (2003).

4 Kiere, L. M., Ramos, A. G. & Drummond, H. No evidence that genetic compatibility drives extra-pair behavior in female blue-footed boobies. *J Avian Biol* **47**, 871-879, doi:10.1111/jav.01061 (2016).

5 Delhey, K., Johnsen, A., Peters, A., Andersson, S. & Kempenaers, B. Paternity analysis reveals opposing selection pressures on crown coloration in the blue tit (Parus caeruleus). *P Roy Soc B-Biol Sci* **270**, 2057-2063, doi:10.1098/rspb.2003.2460 (2003).

6 Foerster, K., Delhey, K., Johnsen, A., Lifjeld, J. T. & Kempenaers, B. Females increase offspring heterozygosity and fitness through extra-pair matings. *Nature* **425**, 714-717, doi:10.1038/nature01969 (2003).

7 Schlicht, L., Girg, A., Loes, P., Valcu, M. & Kempenaers, B. Male extrapair nestlings fledge first. *Anim Behav* **83**, 1335-1343, doi:10.1016/j.anbehav.2012.02.021 (2012).

8 Araya-Ajoy, Y. G. *et al.* Sources of (co)variation in alternative siring routes available to male great tits (Parus major). *Evolution* **70**, 2308-2321, doi:10.1111/evo.13024 (2016).

9 Both, C. *et al.* Delayed age at first breeding and experimental removals show large non-breeding surplus in Pied Flycatchers. *Ardea* **105**, 43-60, doi:10.5253/arde.v105i1.a2 (2017).

10 Bulla, M., Valcu, M., Rutten, A. L. & Kempenaers, B. Biparental incubation patterns in a high-Arctic breeding shorebird: how do pairs divide their duties? *Behav Ecol* **25**, 152-164, doi:10.1093/beheco/art098 (2014).

11 Saladin, V., Ritschard, M., Roulin, A., Bize, P. & Richner, H. Analysis of genetic parentage in the tawny owl (Strix aluco) reveals extra-pair paternity is low. *J Ornithol* **148**, 113-116, doi:10.1007/s10336-006-0109-x (2007).

12 Karell, P., Ahola, K., Karstinen, T., Zolei, A. & Brommer, J. E. Population dynamics in a cyclic environment: consequences of cyclic food abundance on tawny owl reproduction and survival. *J Anim Ecol* **78**, 1050-1062, doi:10.1111/j.1365-2656.2009.01563.x (2009).

13 Brommer, J. E., Karell, P., Aaltonen, E., Ahola, K. & Karstinen, T. Dissecting direct and indirect parental effects on reproduction in a wild bird of prey: dad affects when but not how much. *Behav Ecol Sociobiol* **69**, 293-302, doi:10.1007/s00265-014-1842-4 (2015).

14 Duckworth, R. A. Aggressive behaviour affects selection on morphology by influencing settlement patterns in a passerine bird. *P Roy Soc B-Biol Sci* **273**, 1789-1795, doi:10.1098/rspb.2006.3517 (2006).

15 Duckworth, R. A. & Semenov, G. A. Hybridization Associated with Cycles of Ecological Succession in a Passerine Bird. *Am Nat* **190**, E94-E105, doi:10.1086/693160 (2017).

16 Forstmeier, W., Segelbacher, G., Mueller, J. C. & Kempenaers, B. Genetic variation and differentiation in captive and wild zebra finches (Taeniopygia guttata). *Mol Ecol* **16**, 4039-4050, doi:10.1111/j.1365-294X.2007.03444.x (2007).

17 Schielzeth, H., Burger, C., Bolund, E. & Forstmeier, W. Sexual imprinting on continuous variation: do female zebra finches prefer or avoid unfamiliar sons of their foster parents? J Evolution Biol 21, 1274-1280, doi:10.1111/j.1420-9101.2008.01568.x (2008).

18 Wang, D., Kempenaers, N., Kempenaers, B. & Forstmeier, W. Male zebra finches have limited ability to identify high-fecundity females. *Behav Ecol* **28**, 784-792 (2017).

19 Mathot, K. J., Martin, K., Kempenaers, B. & Forstmeier, W. Basal metabolic rate can evolve independently of morphological and behavioural traits. *Heredity* **111**, 175-181, doi:10.1038/hdy.2013.35 (2013).

20 Wang, D., Forstmeier, W. & Kempenaers, B. No mutual mate choice for quality in zebra finches: Time to question a widely held assumption. *Evolution* **71**, 2661-2676 (2017).

21 Ihle, M., Kempenaers, B. & Forstmeier, W. Fitness Benefits of Mate Choice for Compatibility in a Socially Monogamous Species. *Plos Biol* **13**, e1002248, doi:10.1371/journal.pbio.1002248 (2015).
